# Physical and Functional Constraints on Viable Belowground Acquisition Strategies

**DOI:** 10.3389/fpls.2019.01215

**Published:** 2019-10-11

**Authors:** M. Luke McCormack, Colleen M. Iversen

**Affiliations:** ^1^Center for Tree Science, The Morton Arboretum. Lisle, IL, United States; ^2^Environmental Sciences Division and Climate Change Science Institute, Oak Ridge National Laboratory. Oak Ridge, TN, United States

**Keywords:** fine roots, plant traits, nutrient acquisition, root economics spectrum (RES), mycorrhizal fungi, soil exploration, root lifespan, root diameter

## Abstract

Since their emergence onto land, terrestrial plants have developed diverse strategies to acquire soil resources. However, we lack a framework that adequately captures how these strategies vary among species. Observations from around the world now allow us to quantify the variation observed in commonly-measured fine-root traits but it is unclear how root traits are interrelated and whether they fall along an “economic” spectrum of acquisitive to conservative strategies. We assessed root trait variation and mycorrhizal colonization rates by leveraging the largest global database of fine-root traits (the Fine-Root Ecology Database; FRED). We also developed a heuristic model to explore the role of mycorrhizal fungi in defining belowground exploration efficiency across a gradient of thin- to thick-diameter roots. In support of the expectations of the “root economic spectrum,” we found that root diameter was negatively related to specific root length (Pearson’s *r*=-0.76). However, we found an unexpected negative relationship between root diameter and root tissue density (Pearson’s *r* = -0.40), and we further observed that root nitrogen content was largely unrelated to other economic traits. Mycorrhizal colonization was most closely associated with root diameter (Pearson’s *r* = 0.62) and was unrelated to root tissue density and root nitrogen. The heuristic model demonstrated that while thinner roots have inherently greater capacity to encounter soil resources based on higher surface area per unit mass, the potential for increased associations with mycorrhizal fungi in thicker roots, combined with greater hyphal growth, can result in equally acquisitive strategies for both thin- and thick roots. Taken together, our assessments of root trait variation, trade-offs with mycorrhizal fungi, and broader connections to root longevity allowed us to propose a series of fundamental constraints on belowground resource acquisition strategies. Physical tradeoffs based on root construction (i.e., economic traits) and functional limitations related to the capacity of a root to encounter and acquire soil resources combine to limit the two-dimensional belowground trait space. Within this trait space there remains a diversity of additional variation in root traits that facilitates a wide range of belowground resource acquisition strategies.

## Introduction

Plants depend on roots to perform multiple functions, including anchorage, storage, and most critically, the exploration of soil and acquisition of soil resources by narrow-diameter “fine” roots located at the most distal portions of belowground root systems. Since their emergence onto land over 400 million years ago (Morris et al., 2018), plants have evolved a diverse suites of physical and chemical traits that affect both plant success and terrestrial biogeochemical processes (Raven and Edwards, 2001; Comas et al., 2012; Ma et al., 2018). However, in contrast to the well-understood variation in aboveground plant functional traits (Wright et al., 2004; Díaz et al., 2016), we are only in the early stages of understanding how variation in fine-root traits defines functional plant strategies.

Aboveground, variation in leaf traits is often distilled to an “economic” spectrum of acquisitive to conservative strategies, where cheaply-constructed, fast-growing, and short-lived leaves are considered acquisitive and more resource-intensive and longer-lived leaves are conservative. This “fast-slow” conceptualization has helped to explain leaf trait variation and to link this variation to leaf-level and whole-plant strategies from local to global scales (Westoby and Wright, 2006; Reich, 2014; Díaz et al., 2016; Messier et al., 2017). It should therefore be of little surprise that substantial effort to understand and predict root trait variation has focused on the identification of an equivalent belowground economic spectrum of acquisitive to conservative strategies.

**Figure 1:**
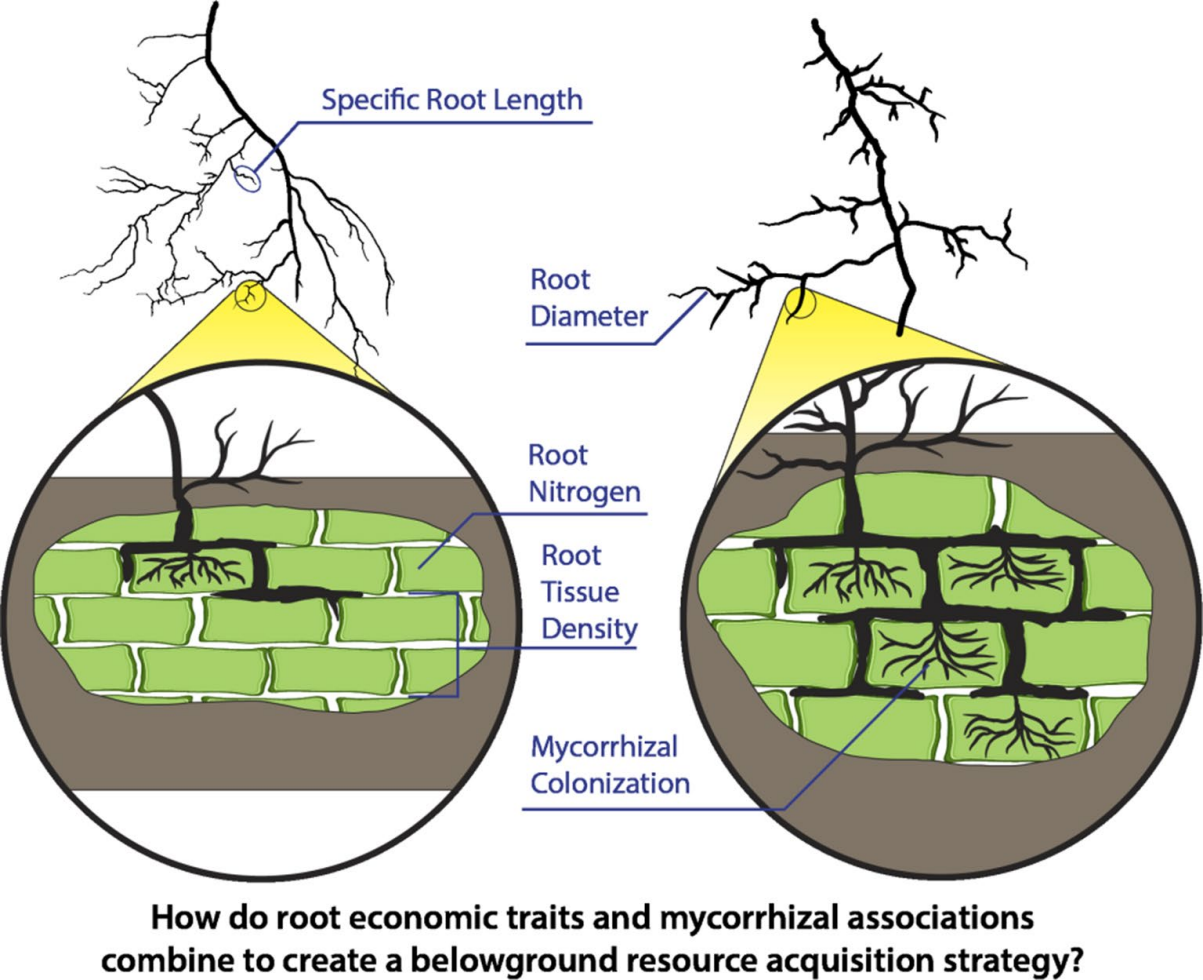
Fine-root economic traits including root diameter, root tissue density, specific root length, and root nitrogen content together with expanded circles portraying differing levels of mycorrhizal associations are depicted in two contrasting root types. Economic traits are commonly expected to define strategies for effective resource acquisition belowground. However, it is unclear how these traits integrate at the level of individual roots (e.g., first-order roots) and how these traits then relate to associations with mycorrhizal fungi which may provide additional resource uptake capacity with increasing rates of colonization and production of external hyphae.

Mirroring the leaf economic spectrum, research belowground has primarily focused on the variation in fine-root morphology and chemistry to understand root functional capacity. As such, aspects of root size and construction, including root diameter, root tissue density (RTD), specific root length (SRL), and measures of nitrogen content have become common traits used to define the functional, i.e., acquisitive, nature of fine roots (**Figure 1**). Cheaply-constructed roots with a thin diameter, low RTD, high SRL, and high nitrogen content are assumed to be more acquisitive, while relatively thick roots with higher tissue densities, lower SRL, and lower nitrogen content are hypothesized to be more conservative (Tjoelker et al., 2005, Reich, 2014; Weemstra et al., 2016).

In recent years, evidence has emerged that both supports and refutes the existence of an acquisitive to conservative spectrum of fine-root traits (Kong et al., 2014; Kramer‐Walter et al., 2016; Roumet et al., 2016; Weemstra et al., 2016). However, we lack a clear explanation for how and why fine-root traits sometimes co-vary and at other times appear independent. Multiple factors likely contribute to confusion in this area including a historical lack of robust root trait data (Iversen et al., 2017), and inconsistent assessments of fine-root traits (McCormack et al., 2015). Additionally, root traits alone may not define functional root strategies in a manner similar to leaves, as the functional role fine roots play in acquiring soil resources is frequently carried out in partnership with mycorrhizal fungi (Smith and Read, 2008; Laliberté, 2017). Finally, there is also the possibility that root traits simply vary in a manner that differs from leaves; either as a result of variation contributed by the mycorrhizal symbiosis or due to the multiple functions carried out by fine roots and the heterogeneous soil environments in which they operate (Bardgett et al., 2014).

Given persistent uncertainties regarding how basic fine-root traits vary within and among species, there is a need to robustly assess whether morphological and chemical fine-root traits are interrelated in a way that is consistent with an acquisitive to conservative trait spectrum. Furthermore, we consider whether a solely root-based assessment of belowground resource acquisition can be used to define plant strategies. While the quality and intensity of associations between roots and mycorrhizal fungi are expected to be highly variable across different root types and ecosystems (Miller et al., 1995; Chen et al., 2016), few have quantified how changing the amounts of mycorrhizal hyphae associated with a given root may fundamentally alter the acquisitive capacity of that root. Taken at the level of individual roots and accounting for both root traits and their mycorrhizal partners, we consider the following conceptual hypothesis: thin- and thick roots, traditionally considered as acquisitive and conservative, respectively, can each express high acquisitive capacities depending on their unique root traits and according to the extent to which they are associated with mycorrhizal fungi. Understanding the relative changes in root reliance on mycorrhizal fungi for soil exploration across functionally divergent root types should be central to our understanding of overall plant strategies for resource acquisition belowground.

Here we use the largest assembly of fine-root trait data to date, the Fine-Root Ecology Database Version 2.0 (FRED; Iversen et al., 2017; McCormack et al., 2018), to analyze global patterns of fine-root trait variation and trait-trait relationships that are expected to define belowground acquisition strategies (**Figure 1**). Specifically, we: 1) test the relationships among core root economic traits (diameter, RTD, SRL, nitrogen content) and 2) assess how these traits vary with mycorrhizal colonization rate. We then use a heuristic model to explore the role of mycorrhizal fungi in defining belowground exploration efficiency across a gradient of thin- to thick-diameter root types. Finally, we integrate our findings to develop a framework for understanding fundamental physical and functional constraints on belowground resource acquisition strategies.

## Methods and Materials

### A Test of Root Economic Trait Relationships Using FRED

Data were collected from FRED version 2.0 which includes over 100,000 root trait observations from roughly 300 trait types from plants across all major vegetated biomes (Iversen et al., 2018). In this analysis, we limited our dataset to include only species-specific root trait observations (i.e., where traits were measured on roots that were identifiable by species, rather than on roots from an unknown mixture of species). We then focused on commonly-measured economic traits considered to be important for defining the acquisitive to conservative spectrum in fine roots including root diameter, root tissue density (RTD), specific root length (SRL), and root nitrogen content. We note that root diameter and SRL are mathematically related and are therefore expected to correlate strongly (Ostonen et al., 2007). We retain both in this analysis to investigate how the strength and patterns of the relationships among these and other economic traits differ. Assessments of mycorrhizal colonization were also included due to the importance of mycorrhizal associations in defining the functional absorptive capacity of a fine-root system. These assessments which identify when mycorrhizal fungal tissues are present on the surface or inside a root provide a first approximation of the relationship and reliance of a plant root system on its fungal partner. Higher colonization rates are generally associated with greater amounts of hyphal length per root length and increased capacity of the joint root-hyphal system to explore the soil (Miller et al., 1995; Treseder, 2013); though it is important to note that associated hyphal length can still vary substantially beyond that predicted by the colonization rate depending on the plant host and soil nutrient conditions (Treseder, 2013; Cheng et al., 2016).

To minimize error associated with different root sampling methodologies, for our analysis of root trait variation we focused on root traits measured on first-order roots (i.e., the most distal roots in the fine-root system) as first-order roots are often the most consistently comparable units within the fine-root branched hierarchy (McCormack et al., 2015). While mycorrhizal colonization is commonly measured on first-order roots for species that associate with ectomycorrhizal fungi, it is more often measured on pooled branches of absorptive fine roots for species that associate with arbuscular mycorrhizal fungi. We used both data types (first-order fine roots and absorptive fine roots) in our analyses of roots associated with arbuscular mycorrhizal fungi as they presented comparable rates of colonization across all plant species. Following assembly of the initial FRED dataset, a series of cleaning steps were taken prior to the final analysis (full details reported in **Text S1**). The final dataset used in this study utilized 1,425 separate observations among 5 traits and encompassed 369 plant species of which 310 were woody and 59 were non-woody. Each individual trait was well-represented with N = 351, 291, 326, 281, and 176 for fine root diameter, RTD, SRL, nitrogen content, and mycorrhizal colonization, respectively. These data represent a diverse, global database encompassing 76 families and 34 taxonomic orders with observations collected across all major, vegetated terrestrial biomes. All data used in this study are freely available in FRED Version 2.0 (Iversen et al., 2018).

Prior to analysis, data for fine-root diameter, RTD, SRL, and nitrogen content were log_10_ transformed to improve the distribution of residuals. The angular transformation was used for mycorrhizal colonization because it is based on a percentage. Trait-trait relationships between each bi-variate trait set were first analyzed using the Pearson correlation (Pearson’s *r*). Statistical results are discussed based on transformed data, though non-transformed data are also presented for clarity and interpretation (Menge et al., 2018). Following assessments of bi-variate correlation, we considered the correlation structure among all traits using principal components analysis (PCA). Analysis was conducted using all species for which data were available from a least two traits (*n* = 369), using Restricted Maximum Likelihood (REML) procedures for bias correction due to gaps within the matrix. Robustness of these results were checked by performing PCA on a smaller dataset where only species for which all traits had been directly measured (i.e., no gaps; *n* = 117) and results were similar (*data not shown*). Pearson correlation analysis was conducted in R version 3.3.2 (R Core Development Team, 2017) and PCA in JMP version 14.0.0 (SAS Institute Inc.). We also conducted additional bi-variate analyses using comparisons between generalized least squares regression (GLS) and phylogenetic generalized least squares regression (PGLS) with the gap-free, 117-species dataset to assess the potential for phylogeny to influence apparent trait coordination among species. The results between GLS and PGLS were generally consistent with the standard correlation analysis and are not presented further in this study.

### Heuristic Model of Belowground Exploration Efficiency

The potential roles of roots and their mycorrhizal partners in co-defining an efficient strategy for belowground soil exploration can be considered using the physical traits of mycorrhizal hyphae together with those of distal, absorptive roots. Considering each component, we may calculate the total absorptive surface area per unit biomass (i.e., specific surface area) attributable to roots alone compared with roots associated with varying amounts of mycorrhizal hyphae. The relative costs and benefits of belowground acquisition efficiency, i.e., specific surface area, varies with the diameter and tissue density of both the root and fungal hyphae as well as with mycorrhizal colonization rate (i.e., percentage of root length colonized by mycorrhizal fungi) and the absolute amount of hyphal length produced per unit of colonized root length. We caution that this model is not intended to present a mechanistic accounting of resource acquisition, nor does it consider factors such as tissue turnover, uptake physiology, or soil physics. Additionally, we do not model potential “exchange rates” between fungal-obtained soil resources and plant derived carbohydrates (for further discussion see Brzostek et al., 2014). Instead the model is focused on the central theme of whether a pattern of acquisitive to conservative uptake strategies, defined by having greater to lesser absorptive surface area per unit root mass, exists along a gradient of thin- to thick-diameter fine roots as predicted by the “fast-slow” economics spectrum, or whether associations with mycorrhizal fungi influence the expected pattern.

The heuristic model explicitly represents variation in potential mycorrhizal root absorptive surface area based on relationships between root diameter, RTD, and colonization by mycorrhizal fungi at the level of distal, absorptive roots. Parameter estimates for biomass costs and potential benefits for uptake in terms of absorptive surface area are obtained from empirical trait relationships observed within FRED (see below and **Text S2**) and on a synthesis of additional literature values to determine hyphal parameters. We focused our model parameterization of root and fungal traits on plants that were either non-mycorrhizal or associated with arbuscular mycorrhizal fungi because i) fine-root traits and trait relationships are likely to vary based on the type of mycorrhizal association (e.g., arbuscular mycorrhizal, ectomycorrhizal, ericoid mycorrhizal fungi), and ii) most reports on hyphal production rates were derived from plant systems dominated by arbuscular mycorrhizal fungi. Furthermore, arbuscular mycorrhizal fungi are obligate symbionts with the plant root, which serves as the sole source carbohydrates to the fungus and non-mycorrhizal and AM associates together represent roughly 85% of known plant species (Brundrett and Tedersoo, 2018). Using only non-mycorrhizal or arbuscular mycorrhizal plant species, we re-analyzed the relationships among first-order root diameter, RTD, and absorptive root mycorrhizal colonization rates (**Figure S1**). These results were then used to parameterize the heuristic model (**Text S2**).

Six model scenarios are presented with the heuristic model (**Table S1**). We first established a scenario where mycorrhizal fungi are not considered and belowground absorptive surface area is determined solely by root traits (“roots only”). Then we add four scenarios considering the range of traits associated with mycorrhizal fungi including low- and high hyphal production rates (1 and 10 m of hyphal length per cm colonized root length, respectively) as well as small and large hyphal diameters (3 and 10 µm, respectively). These mycorrhizal trait ranges were selected to encompass lower and higher values previously reported (**Text S2**), but neither the high nor the low values necessarily represent the “average” condition, which instead likely falls somewhere in between. In an effort to approximate a potential average condition, we added an additional scenario based on the assumption that hyphal production rates will likely increase with increasing average root diameter (“gradient scenario”). To approximate a gradient of increased reliance on mycorrhizal fungi in thicker-diameter roots, we allowed the hyphal production rate to increase linearly from small- to large-diameter roots (**Figure S1**, **Table S1**). A linear trend was used as a first approximation for this relationship, though it is possible that this would vary based on additional factors in natural systems including aspects of root anatomy and physiology as well as soil fertility. For the gradient scenario we used an average hyphal diameter of 6.5 µm.

## Results

### Test of Root Economic Trait Relationships Using FRED

**Figure 2:**
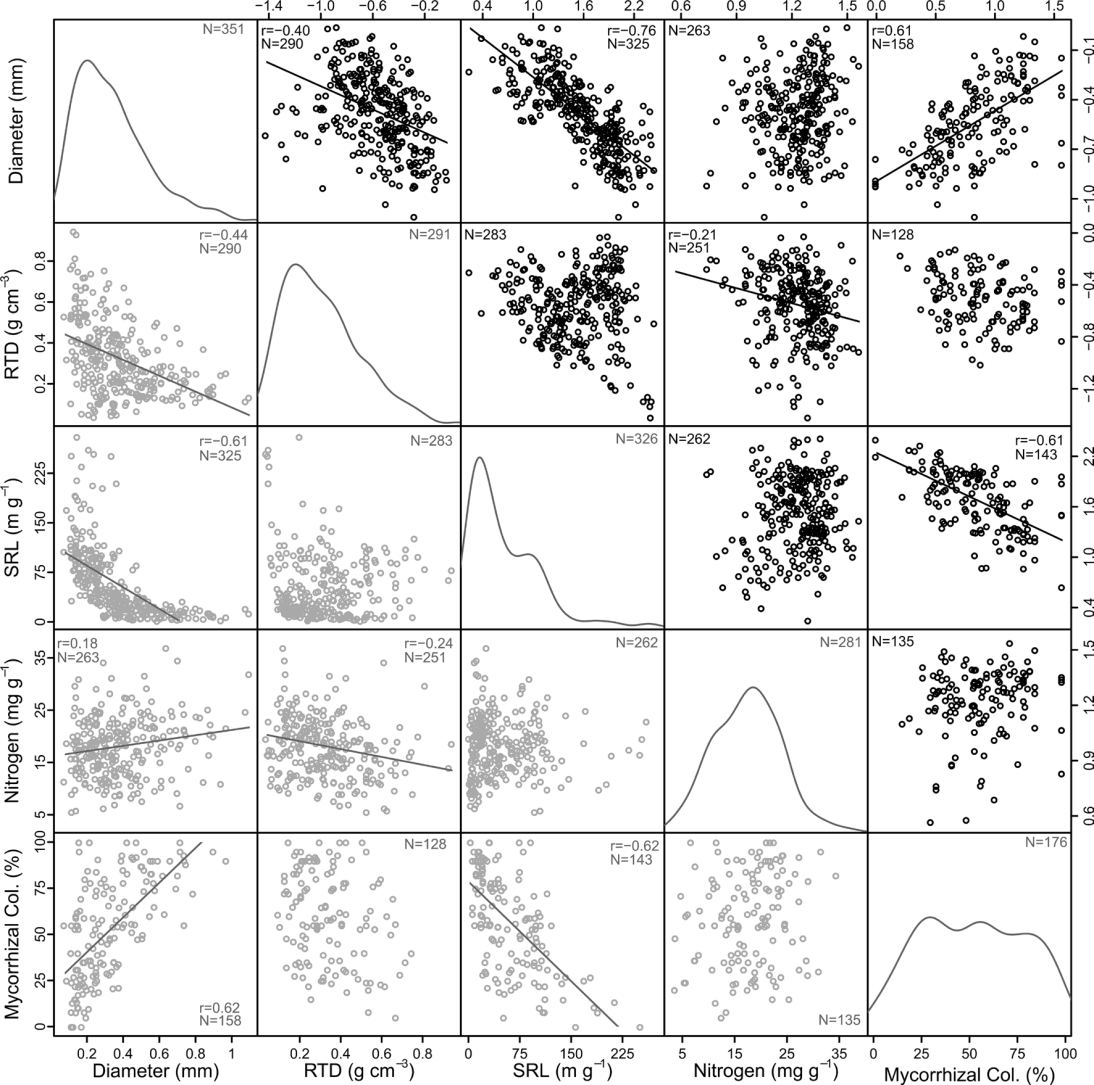
Correlations among diameter, root tissue density (RTD), specific root length (SRL), and nitrogen content of first-order roots and mycorrhizal colonization rate (Mycorrhizal Col.). Linear fits and Pearson’s correlation coefficient (*r*) are shown for significant relationships (*p* ≤ 0.05) along with sample numbers (*N*). Note that quadratic fits better capture non-transformed data in some cases but do not change the observed trends. Black circles in the upper diagonal show transformed data and gray circles in the lower diagonal are non-transformed. Kernel density plots show the distribution of non-transformed data.

Assessments of bivariate relationships among basic morphological traits and nitrogen content of first-order roots revealed patterns that were both consistent and inconsistent with a traditional economics spectrum (**Figure 2**). As expected, root diameter was strongly and negatively related to specific root length (SRL, *r* = -0.76). However, root diameter was also negatively related to root tissue density (RTD), resulting in lower densities on average among thicker roots compared to thinner roots (*r* = -0.40; **Figure 2**). It is important to note, however, that among the thinner roots there was also substantial variation with some having relatively high, and others relatively low RTD (see non-transformed presentation in **Figure 2** and **Figure S1b**). Correlations between the morphological traits and root nitrogen content were consistently weak with the only significant result being a negative relationship between RTD and nitrogen content (*r* = -0.21), though there was also a weakly positive relationship between diameter and nitrogen content in the non-transformed data (*r* = 0.18; **Figure 2**). Mycorrhizal colonization was strongly and positively related to root diameter (*r* = 0.62) and negatively related to SRL (*r* = -0.61), though in both cases this was most consistent among species associating with arbuscular mycorrhizal fungi and not for ectomycorrhizal associates (**Figure S2**). In contrast, mycorrhizal colonization was only weakly related to RTD (*r* = -0.23; **Figure 2**). There was no relationship between mycorrhizal colonization and root nitrogen content.

**Figure 3:**
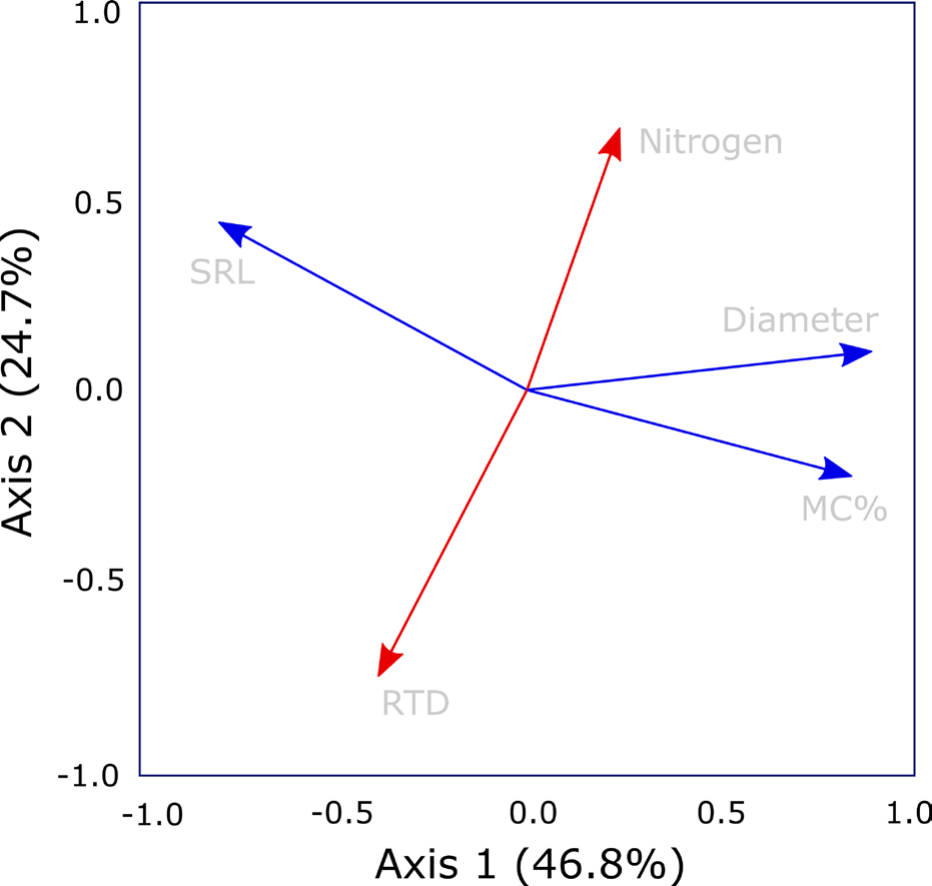
Principal components analysis of core root economic traits; root diameter, root tissue density (RTD), specific root length (SRL), and root nitrogen, all measured on first-order roots, together with mycorrhizal colonization rate. The analysis suggests departure from a singular root economics spectrum as root nitrogen and RTD (highlighted in red) are orthogonal to Axis 1 (highlighted in blue) which is defined by diameter, SRL and mycorrhizal colonization rate. Trait loadings for PC 1, 2, and 3 are given in **Table 1**.

The PCA demonstrated that the five traits assessed in this study arrayed themselves along multiple axes rather than a single axis of root economic strategies (**Figure 3**). The primary variables related to construction aligned on separate axes, with diameter closely associated with the first axis and RTD aligned on the second axis (**Table 1**). Specific root length was associated with both axes 1 and 2, but was more closely associated with axis 1. Additionally, mycorrhizal colonization was well-aligned with diameter on axis 1 while root nitrogen was most closely associated with the second axis opposite RTD. The first axis of the PCA explained 49.5% of the variation and had the strongest loadings for root diameter, mycorrhizal colonization, and SRL while the second axis captured 23% of the variation and was most closely related RTD and root nitrogen.

**Table 1:** Trait loadings for principal components analysis (displayed in **Figure 3**). The percentage of variation explained is listed with each axis. Loadings listed in bold were considered to be significant (*p* < 0.05).

Trait	PC 1 (46.1%)	PC 2 (25.4%)	PC 3 (16.4%)
Nitrogen	0.225	**0.648**	0.727
Diameter	**0.896**	0.137	–0.142
Root tissue density (RTD)	–0.324	–**0.780**	**0.504**
Specific root length (SRL)	**–0.801**	**0.428**	–0.125
Mycorrhizal Colonization	**0.842**	–0.203	–0.025

### Belowground Exploration Efficiency Across Root Types

**Figure 4:**
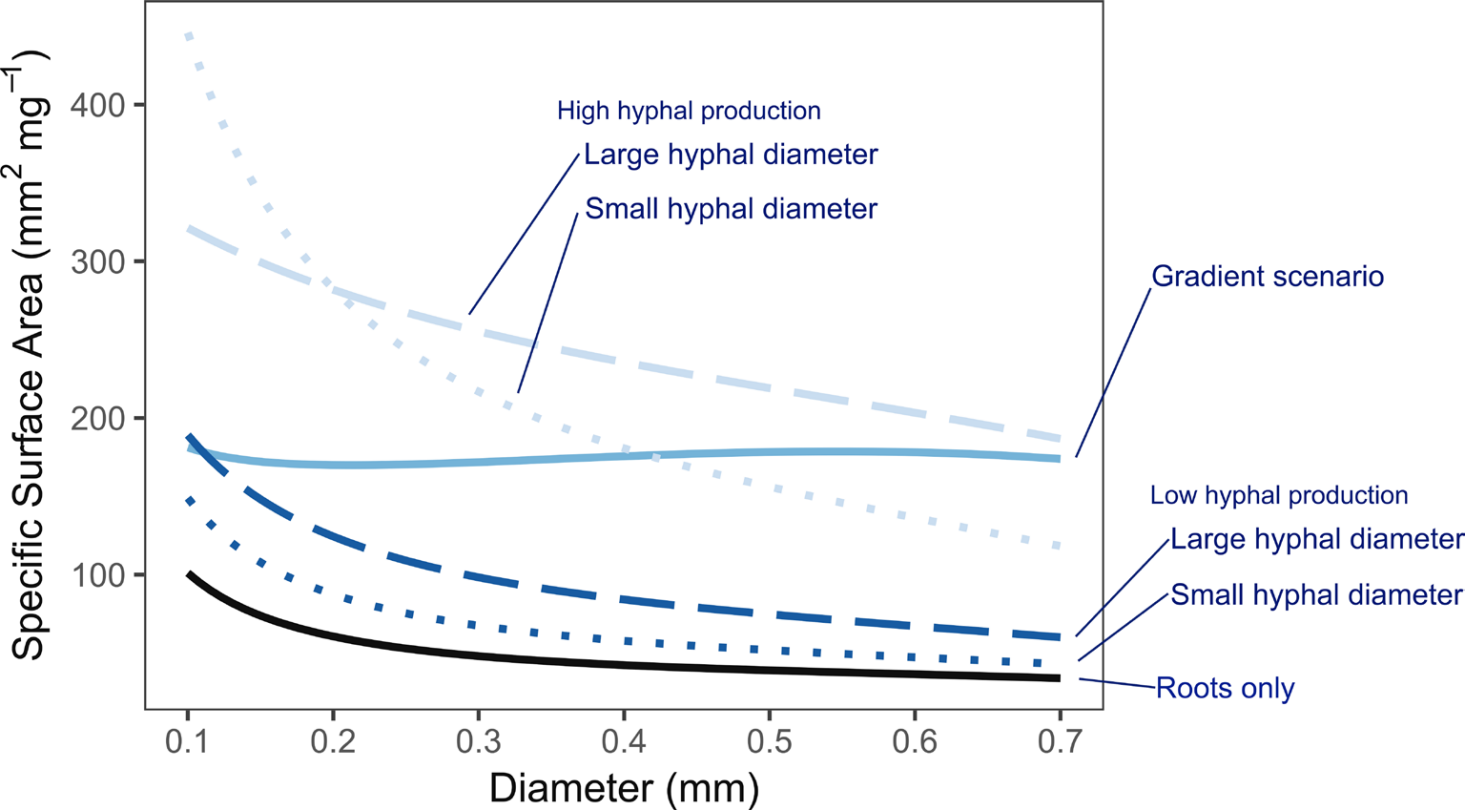
Heuristic model of belowground exploration efficiency based on the specific surface area of roots only (solid black line), and roots combined with mycorrhizal hyphae (blue lines). Low vs. high hyphal production rates are indicated by dark and light blue lines, respectively. Small vs. large hyphal diameters are indicated by dotted and dashed lines, respectively. The gradient scenario where hyphal production rates increase with root diameter is shown in the solid, medium blue line. Scenarios with hyphal production consistently have higher exploration efficiency than roots alone with higher hyphal production rates showing greater increases than low production rates. With roots alone and under fixed hyphal production scenarios (high and low), thinner roots consistently had greater exploration efficiency. This advantage was lost under the gradient scenario which suggests greater parity across the diameter range.

The heuristic model focuses on tradeoffs between construction costs and potential benefit from absorptive surface area. While the concepts may be generalizable, the model is explicitly parameterized to represent distal, absorptive fine roots and the arbuscular mycorrhizal symbiosis. The model clearly highlights that the potential surface area per unit mass (i.e., specific surface area) provided by mycorrhizal hyphae is much greater than by the roots themselves. This was true across the gradient of thin- to thick-diameter roots, though less dramatic for scenarios assuming low rates of hyphal production (i.e., 1 m vs. 10 m hyphae per cm colonized root) and smaller-diameter hyphae (i.e., 3 µm vs. 10 µm). Overall, the potential exploration efficiency increases by 2- to 6-fold when considering both roots and fungi together over roots alone (**Figure 4**). Depending on the scenario, the model also indicates that plants may achieve an “acquisitive” rooting strategy by constructing either thin-diameter roots, or in contrast, thicker-diameter roots that associate with mycorrhizal fungi. According to the scenarios where hyphal production rates were fixed across all root diameters, the estimated surface area per unit mass decreases from the thinnest to the thickest roots, supporting the idea that thin roots are more acquisitive even when accounting for mycorrhizal fungi. However, when hyphal production rates are allowed to increase with increasing diameter as might be expected given the greater volume of root cortex available for mycorrhizal colonization in thicker roots, the model predicts nearly equivalent acquisitive capacities across the range of root diameters considered. This is in contrast to predictions of an acquisitive to conservative spectrum based on root economic traits.

Changing from a fixed hyphal production rate to a gradient scenario of increasing mycorrhizal colonization with increasing root diameter fundamentally changed the interpreted result. Rather than more acquisitive thinner roots, there was greater parity among thin- and thick-rooted strategies. The wide range of estimates used in this model are reflective of high variability and uncertainty due to limited observations in the field. Yet, the large differences in model predictions between scenarios with high- or low hyphal production rates indicate that this as an especially important parameter needed to estimate the explorative and potential absorptive capacity of a fine-root system. Therefore, increased measures of hyphal length and biomass associated with fine roots across a range of plant species, root morphologies, and environmental contexts are much needed.

## Discussion

### Fine-Root Economics and Functional Constraints

Central to describing functional plant strategies at both local and global scales has been a need to assess whether fine-root traits vary in a predictable manner. Reflecting understanding aboveground, interpretations of belowground trait variation have focused on the idea of coordinated trait variation among core root economic traits related to investments in the construction of fine roots; namely root diameter, specific root length (SRL), root tissue density (RTD), and nitrogen content. Our global analysis of these four traits highlights correspondence with, and notable deviations from, a spectrum of acquisitive to conservative belowground strategies. These apparently mixed results are generally in agreement with other regional and large-scale analyses of root trait variation (Chen et al., 2013, Kong et al., 2014; Ma et al., 2018). The negative relationship we observed between root diameter and RTD, which is not consistent with the “fast-slow” economics spectrum, coupled with a positive relationship between diameter and mycorrhizal associations makes it possible for thick, large-diameter roots to express a relatively acquisitive strategy despite being traditionally defined as conservative. This finding was supported by a heuristic model, which highlighted the acquisitive capacity inherent to relatively thin roots while also demonstrating how this capacity may be matched by thicker roots with greater reliance on mycorrhizal fungi. Moreover, results from the heuristic model should serve as a strong reminder that defining the acquisitive capacity of a root system based solely on root traits is likely to miss a large proportion of the acquisitive surface area leveraged by a plant to explore soil volumes. The complementary nature of root traits and associations with mycorrhizal fungi facilitates a diversity of functionally acquisitive strategies for uptake of belowground resources across the spectrum of thin- to thick fine roots.

The overall return on investment for a fine-root strategy depends partly on the standing biomass capable of acquiring belowground resources, as emphasized by our heuristic model, as well as the longevity and turnover of those acquisitive tissues. Average lifespans of absorptive roots tend to vary widely across species, with estimates generally ranging from several months to a few years (McCormack et al., 2012; Liu et al., 2016). Consistent with an economics framework, fine-root diameter and RTD are often positively, and SRL negatively, related to fine-root lifespan, though diameter seems to be more important among woody species while RTD may be a better predictor among herbaceous species (Ryser, 1996; McCormack et al., 2012; Adams et al., 2013, Sun et al., 2016). Independent of morphological traits, fine-root nitrogen content is also negatively related to fine-root lifespan (Tjoelker et al., 2005; Withington et al., 2006; McCormack et al., 2012; Sun et al., 2016). However, the independence among many root economic traits results in the potential for relatively long-lived fine-roots to be the result of changes in root size (e.g., larger diameters) while in other cases longer lifespans may occur in smaller roots driven by high RTD and/or low nitrogen content. While less is known about the variation in lifespan among fungal hyphae, estimates for their individual lifespans are consistently shorter and tend to range from days to several weeks (Staddon et al., 2003; Godbold et al., 2006; Allen and Kitajima, 2013).

Established relationships between economic traits and fine-root longevity clearly indicate that basic patterns of investment in root tissue construction are an important component in defining a fine-root strategy. However, limited coordination among economic traits and an important role of mycorrhizal fungi suggest that our understanding of belowground resource acquisition strategies should move beyond root economic traits. Here we propose a set of fundamental constraints on fine-root strategies for belowground resource capture that broadly encompass the global trait space occupied by terrestrial plants (**Box 1**). These include physical constraints which limit what types of roots may realistically be constructed (**Box 1a, b**) as well as functional constraints that limit the competitive ability of a fine-root strategy even if it is physically possible to construct (**Box 1c, d**). Given that most terrestrial plants must express a viable strategy for soil resource acquisition, this framework should be generalizable to the vast majority of terrestrial plants. However, there are additional fine-root, mycorrhizal, and whole-plant adaptations that serve as important determinants of belowground strategies that may, in many cases, be independent of these initial trait dimensions. We discuss these further below.

Box 1 **Fundamental constraints on viable fine-root strategies for resource acquisition.**

**Figure:**
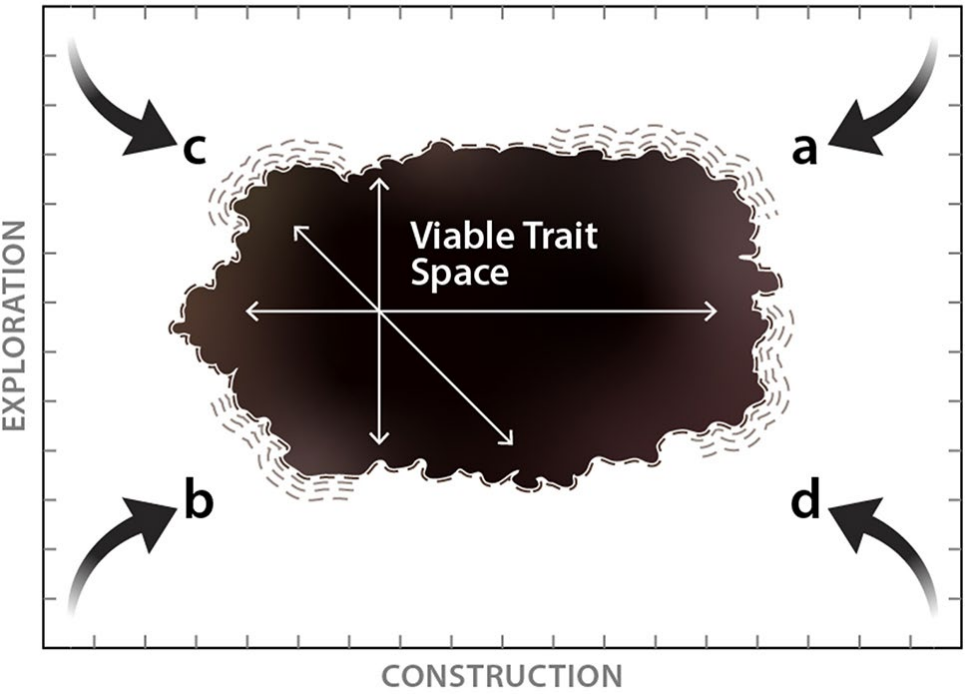

The x-axis is related to the root economic spectrum and is defined by increasing investment into the individual fine root. Moving from left to right, this investment is primarily captured by increasingly thick-diameter roots and/or greater investment in root tissue density (RTD) as well as increased reliance on mycorrhizal fungi and greater root longevity. The y-axis represents potential for roots to explore and encounter soil resources. Moving from bottom to top, increasing exploration capacity is expressed as increasing surface area from root and mycorrhizal tissues available to encounter soil resources divided by the total mass invested in those tissues (i.e., the specific surface area).

**Physical Constraints** of impossible belowground strategies due to trade-offs in size, mass, density:

**a –** Trait space in the upper-right corner is unoccupied as it is not possible to construct roots with very large diameters or extremely high tissue density that also have a high absorptive potential as potential gains from associations with mycorrhizal fungi are eventually diminished by lower specific surface area of the root.
**b –** Trait space in the lower-left corner is unoccupied as it is not physically possible to construct a very thin root, or a root with extremely low tissue density, that also has low exploration efficiency. This is due to the high surface area to volume ratios of very thin roots and the low biomass cost of lower tissue density.

**Functional Constraints** inhibit strategies that can physically exist but are often impractical:

**c –** While it may be possible to construct narrow-diameter fine roots with increasingly low tissue density, there would likely be trade-offs that result in short root lifespans and excessively high root population turnover rates. This pushes viable trait space inward from the upper-left corner though aspects of this strategy may occur in species with very short life cycles or in cases where sparse resource availability favors high specific surface area in spite of short lifespans (e.g., rain roots, cluster roots; Nobel et al., 1990, Teste et al., 2018).
**d –** Fine roots with increasingly large diameter or very high tissue density may be accompanied by increasingly long lifespans while plants may further conserve resources by limiting allocation to mycorrhizal fungi. However, the very low specific surface area of these roots would make this strategy non-competitive in most cases and pushes viable trait space inward from the bottom-right corner.

### Diverse Fine-Root Strategies Exist Within Physical and Functional Constraints

The multi-dimensional trait space available within the constraints presented in **Box 1** represents the flexibility in how plants may acquire limiting resources. This is critically important because fine roots are responsible for the capture of multiple resources, including mobile and immobile plant nutrients as well as water. Strategies that increase the total belowground absorptive surface area per unit mass are thought to be more effective at increasing the potential of a root system to encounter relatively immobile resources. As indicated by our heuristic model, this may be accomplished *via* changes in root construction as well as through associations with mycorrhizal fungi. Additional mechanisms not considered in our model may also be employed to increase the absorptive surface area of fine roots. At the individual root level, the profusion of root hairs can roughly double the surface area of a cylindrical root and provide an alternative to investment in mycorrhizal fungi (Schweiger et al., 1995; Jungk, 2001; Leitner et al., 2010). Associations with other microbial symbionts can also be critical, especially those formed with nitrogen-fixing bacteria which represents an important nitrogen acquisition strategy for many plants (Vitousek et al., 2013). Adjustments at the root system level, including shifts in fine-root biomass, fine-root branching architecture, and rooting depth distribution can also have a significant impact on the ability of plants to explore and encounter resources belowground. (Fitter, 1987; Iversen et al., 2008; Freschet et al., 2015). Importantly, these changes can be independent of root morphology and chemistry, facilitating greater intra- and interspecific adaptation to different environments (Li et al., 2017; Liese et al., 2017).

Physiological changes may also be used to increase resource uptake and add further trait dimensions for belowground resource acquisition. For example, root exudates can facilitate the release of nutrients adsorbed to soil minerals or be used to stimulate the activity of soil microbes and increase mineralization rates and access to nutrients complexed with organic matter (Gardner et al., 1983; Lopez-Bucio et al., 2000; Dakora and Phillips, 2002; Phillips et al., 2011; Bengtson et al., 2012). Plants can also affect greater rates of resource uptake by “pulling” more resources to the root surface by upregulating transporter activity, altering nutrient uptake kinetics, and creating steeper concentration gradients that then increase rates of solute diffusion (e.g., Zhu et al., 2016). At the scale of the whole plant, transpiration streams pulling water through bulk soil may also be an important mode of acquiring soil resources that requires little to no adjustment belowground (McMurtrie and Näsholm, 2018), though this would only be relevant for available water and resources that are sufficiently mobile in soil solutions (e.g., NO_3_^-^).

As emphasized in our heuristic model, mycorrhizal fungi can also be important contributors to the acquisition of belowground resources. While our heuristic model focused on differences in hyphal production among root types, it is important to point out that colonization by different species of mycorrhizal fungi may also result in changes in extraradical hyphal production (Agerer, 2001, Hart and Reader, 2002b). Furthermore, mycorrhizal fungi may enhance resource uptake through a number of additional pathways that go beyond intra- and interspecific changes in hyphal production. The smaller diameter of fungal hyphae compared to roots enable them to access resources in smaller pore spaces that would be otherwise be inaccessible. Mycorrhizal fungi utilize exudates, and in some cases release enzymes to prime microbial communities or directly catalyze the breakdown of organic matter (Cheng et al., 2012; Lindahl and Tunlid, 2015; Meier et al., 2015; Talbot et al., 2015). Some fungi also acquire limiting nutrients through the release of low molecular weight acids and direct mineral weathering (Van Breemen et al., 2000; Taylor et al., 2009). Increases in root stress tolerance, pathogen resistance, and extended root persistence resulting from some mycorrhizal associations can also help maintain a root system with less frequent replacement (Marx, 1972; Azcón-Aguilar and Barea, 1997; Schutzendubel and Polle, 2002; Evelin et al., 2009; Fernandez et al., 2013). While our heuristic model explicitly focused on associations with arbuscular mycorrhizal fungi, we suspect that associations with different types of mycorrhizal fungi (e.g., ectomycorrhizal, ericoid mycorrhizal, and others) are likely accompanied by unique changes in fine-root construction and root trait relationships and should therefore be an important focus of future studies.

Finally, it is important to consider that many aspects of belowground strategies and variation in those strategies may not be the result of strict optimization for acquisition of limiting resources by either the plant or the mycorrhizal fungi. First, optimization for one root function may in some cases compromise another. For example, increasing the proportion of stele within a fine root can increase mechanical strength and plant anchorage but it will also result in reduced cortex area (Mao et al., 2018), potentially reducing association with mycorrhizal fungi. Second, belowground acquisition strategies are partly shaped by competition with neighbors in ways that may not be appear optimal for an individual plant (Gersani et al., 2001; Dybzinski et al., 2019). Lastly, associations with mycorrhizal fungi represent a significant cost to the plant and the overall benefit to the plant host varies among species and with environmental context (Wilson and Hartnett, 1998, Hart and Reader, 2002a; Hoeksema et al., 2010; Ekblad et al., 2013). While our heuristic model implicitly assumes equivalence between one unit of root or fungal surface area, it is important to acknowledge that resource exchange does not necessarily occur in a “one-to-one” manner (Brzostek et al., 2014). Indeed, fungal tissues may retain significant amounts of limiting nutrients in their tissues, exacerbating rather than alleviating nutrient limitation for plants in some contexts (Näsholm et al., 2013; Baskaran et al., 2017). As a result, trade-offs between roots and mycorrhizal fungi are likely to be complex as both partners seek to maximize benefits under limiting conditions. Taken to their extreme, these trade-offs may result in declines in mycorrhizal associations in favor of non-mycorrhizal plant strategies in severely nutrient-impoverished ecosystems (Zemunik et al., 2015; Raven et al., 2018; Zemunik et al., 2018).

### Conclusions

We combined an analysis of fine-root trait observations from around the world (FRED) with a heuristic model of belowground exploration at the level of first-order roots to assess how basic root economic traits are interrelated and how variation in these traits and associations with mycorrhizal fungi likely alter belowground strategies. Our analysis, together with emerging evidence from other studies, lead us to the conclusion that an economics framework remains a useful starting point for understanding variation in resource investment and persistence across root strategies, but it cannot remain an endpoint. Wider discussions of root function, and soil resource acquisition in particular, must move beyond the traditional acquisitive to conservative spectrum often inferred by root economic traits and should actively consider the role of mycorrhizal fungi. Future efforts to integrate additional traits including root architecture, multiple aspects of root physiology, and root dynamics will also be invaluable. The multi-functional nature of fine roots and the dynamic soil environment in which roots exist make it likely that few plants express an optimal belowground strategy for any one function and instead engage in a bet-hedging strategy where a single root should perform well at multiple functions, though not optimal for any single activity.

Given the important, yet poorly-quantified role, of mycorrhizal associations, there is a strong need to invest in field measurements of fine-root processes coupled with mycorrhizal traits that define their capacity to aid in plant resource capture. Contemporary efforts to model root and mycorrhizal activities at the scale of individual roots and up to the global terrestrial environment continue to emphasize the broad importance of this symbiosis, its impact on plant nutrition, and its potential global consequences (Yanai et al., 1995; Orwin et al., 2011; Brzostek et al., 2014; Deckmyn et al., 2014; Baskaran et al., 2017). It is therefore imperative that as we continue to search for broad trait relationships, especially belowground, we work to account for the role of mycorrhizal associations in shaping functional plant strategies.

## Data Availability Statement

The datasets generated for for this study are freely available through the Fine-Root Ecology Database (FRED; https://roots.ornl.gov/) and available on request to the corresponding author.

## Author Contributions

MM and CI conceived the study. MM conducted the analyses and wrote the manuscript with CI.

## Funding

MM and CI were supported by the Office of Biological and Environmental Research in the United States Department of Energy’s Office of Science. This manuscript has been authored by UT-Battelle, LLC under Contract No. DE-AC05-00OR22725 with the U.S. Department of Energy.

## Conflict of Interest

The authors declare that the research was conducted in the absence of any commercial or financial relationships that could be construed as a potential conflict of interest.

## References

[B1] Adams T. S. McCormack M. L. Eissenstat D. M. (2013). Foraging strategies in trees of different root morphology: the role of root lifespan. Tree Physiol. 33, 940–948. 10.1093/treephys/tpt067 24128849

[B2] Agerer R. (2001). Exploration types of ectomycorrhizae. Mycorrhiza 11, 107–114. 10.1007/s005720100108

[B3] Allen M. F. Kitajima K. (2013). In situ high-frequency observations of mycorrhizas. New Phytol. 200, 222–228. 10.1111/nph.12363 23772913

[B4] Azcón-Aguilar C. Barea J. (1997). Arbuscular mycorrhizas and biological control of soil-borne plant pathogens–an overview of the mechanisms involved. Mycorrhiza 6, 457–464. 10.1007/s005720050147

[B5] Bardgett R. D. Mommer L. De Vries F. T. (2014). Going underground: root traits as drivers of ecosystem processes. Trends Ecol. Evol. 29, 692–699. 10.1016/j.tree.2014.10.006 25459399

[B6] Baskaran P. Hyvönen R. Berglund S. L. Clemmensen K. E. Ågren G. I. Lindahl B. D. (2017). Modelling the influence of ectomycorrhizal decomposition on plant nutrition and soil carbon sequestration in boreal forest ecosystems. New Phytol. 213, 1452–1465. 10.1111/nph.14213 27748949

[B7] Bengtson P. Barker J. Grayston S. J. (2012). Evidence of a strong coupling between root exudation, C and N availability, and stimulated SOM decomposition caused by rhizosphere priming effects. Ecol. Evol. 2, 1843–1852. 10.1002/ece3.311 22957187 PMC3433989

[B8] Brundrett M. C. Tedersoo L. (2018). Evolutionary history of mycorrhizal symbioses and global host plant diversity. New Phytol. 220, 1108–1115. 10.1111/nph.14976 29355963

[B9] Brzostek E. R. Fisher J. B. Phillips R. P. (2014). Modeling the carbon cost of plant nitrogen acquisition: Mycorrhizal trade-offs and multipath resistance uptake improve predictions of retranslocation. J. Geophys. Res. Biogeosci. 119, 1684–1697. 10.1002/2014JG002660

[B10] Chen W. Koide R. T. Adams T. S. DeForest J. L. Cheng L. Eissenstat D. M. (2016). Root morphology and mycorrhizal symbioses together shape nutrient foraging strategies of temperate trees. Proc. Natl. Acad. Sci. U. S. A 113, 8741–8746. 10.1073/pnas.1601006113 27432986 PMC4978252

[B11] Chen W. Zeng H. Eissenstat D. M. Guo D. (2013). Variation of first-order root traits across climatic gradients and evolutionary trends in geological time. Global Ecol. Biogeogr. 22, 846–856. 10.1111/geb.12048

[B12] Cheng L. Booker F. L. Tu C. Burkey K. O. Zhou L. Shew H. D. (2012). Arbuscular mycorrhizal fungi increase organic carbon decomposition under elevated CO2. Science 337, 1084–1087. 10.1126/science.1224304 22936776

[B13] Cheng L. Chen W. Adams T. S. Wei X. Li L. McCormack M. L. (2016). Mycorrhizal fungi and roots are complementary in foraging within nutrient patches. Ecology 97, 2815–2823. 10.1002/ecy.1514 27859112

[B14] Comas L. Mueller K. Taylor L. Midford P. Callahan H. Beerling D. (2012). Evolutionary patterns and biogeochemical significance of angiosperm root traits. Int. J. Plant Sci. 173, 584–595. 10.1086/665823

[B15] Dakora F. D. Phillips D. A. (2002). Root exudates as mediators of mineral acquisition in low-nutrient environments. Plant Soil 245, 35–47. 10.1023/A:1020809400075

[B16] Deckmyn G. Meyer A. Smits M. M. Ekblad A. Grebenc T. Komarov A. (2014). Simulating ectomycorrhizal fungi and their role in carbon and nitrogen cycling in forest ecosystems. Can. J. Forest Res. 44, 535–553. 10.1139/cjfr-2013-0496

[B17] Díaz S. Kattge J. Cornelissen J. H. Wright I. J. Lavorel S. Dray S. (2016). The global spectrum of plant form and function. Nature 529, 167–171. 10.1038/nature16489 26700811

[B18] Dybzinski R. Kelvakis A. McCabe J. Panock S. Anuchitlertchon K. Vasarhelyi L. (2019). How are nitrogen availability, fine-root mass, and nitrogen uptake related empirically? Implications for models and theory. Glob. Chang. Biol. 25, 885–899. 10.1111/gcb.14541 30536492

[B19] Ekblad A. Wallander H. Godbold D. Cruz C. Johnson D. Baldrian P. (2013). The production and turnover of extramatrical mycelium of ectomycorrhizal fungi in forest soils: role in carbon cycling. Plant Soil 366, 1–27. 10.1007/s11104-013-1630-3

[B20] Evelin H. Kapoor R. Giri B. (2009). Arbuscular mycorrhizal fungi in alleviation of salt stress: a review. Ann. Bot. 104, 1263–1280. 10.1093/aob/mcp251 19815570 PMC2778396

[B21] Fernandez C. W. McCormack M. L. Hill J. M. Pritchard S. G. Koide R. T. (2013). On the persistence of Cenococcum geophilum ectomycorrhizas and its implications for forest carbon and nutrient cycles. Soil Biol. Biochem. 65, 141–143. 10.1016/j.soilbio.2013.05.022

[B22] Fitter A. H. (1987). An architectural approach to the comparative ecology of plant root systems. New Phytol. 106, 61–77. 10.1111/j.1469-8137.1987.tb04683.x

[B23] Freschet G. T. Swart E. M. Cornelissen J. H. (2015). Integrated plant phenotypic responses to contrasting above-and below-ground resources: key roles of specific leaf area and root mass fraction. New Phytol. 206, 1247–1260. 10.1111/nph.13352 25783781

[B24] Gardner W. Barber D. Parbery D. (1983). The acquisition of phosphorus byLupinus albus L. Plant Soil 70, 107–124. 10.1007/BF02374754

[B25] Gersani M. O’Brien E. E. Maina G. M. Abramsky Z. (2001). Tragedy of the commons as a result of root competition. J. Ecol. 89, 660–669. 10.1046/j.0022-0477.2001.00609.x

[B26] Godbold D. L. Hoosbeek M. R. Lukac M. Cotrufo M. F. Janssens I. A. Ceulemans R. (2006). Mycorrhizal hyphal turnover as a dominant process for carbon input into soil organic matter. Plant Soil 281, 15–24. 10.1007/s11104-005-3701-6

[B27] Hart M. M. Reader R. J. (2002a). Host plant benefit from association with arbuscular mycorrhizal fungi: variation due to differences in size of mycelium. Biol. Fertil. Soils 36, 357–366. 10.1007/s00374-002-0539-4

[B28] Hart M. M. Reader R. J. (2002b). Taxonomic basis for variation in the colonization strategy of arbuscular mycorrhizal fungi. New Phytol. 153, 335–344. 10.1046/j.0028-646X.2001.00312.x

[B29] Hoeksema J. D. Chaudhary V. B. Gehring C. A. Johnson N. C. Karst J. Koide R. T. (2010). A meta-analysis of context-dependency in plant response to inoculation with mycorrhizal fungi. Ecol. Lett. 13, 394–407. 10.1111/j.1461-0248.2009.01430.x 20100237

[B30] Iversen C. M. Ledford J. Norby R. J. (2008). CO2 enrichment increases carbon and nitrogen input from fine roots in a deciduous forest. New Phytol. 179, 837–847. 10.1111/j.1469-8137.2008.02516.x 18537885

[B31] Iversen C. M. McCormack M. L. Powell A. S. Blackwood C. B. Freschet G. T. Kattge J. (2017). A global Fine-Root Ecology Database to address below-ground challenges in plant ecology. New Phytol. 215, 15–26. 10.1111/nph.14486 28245064

[B32] Iversen C. M. Powell A. McCormack M. Blackwood C. Freschet G. Kattge J. (2018). Fine-Root Ecology Database (FRED): a global collection of root trait data with coincident site, vegetation, edaphic, and climatic data, Version 2. ORNLTESSFA (Oak Ridge National Lab’s Terrestrial Ecosystem Science Scientific Focus Area (ORNL TES SFA)).

[B33] Jungk A. (2001). Root hairs and the acquisition of plant nutrients from soil. J Plant Nutr Soil Sci (1999) 164, 121–129. 10.1002/1522-2624(200104)164:2<121::AID-JPLN121>3.0.CO;2-6

[B34] Kong D. L. Ma C. G. Zhang Q. Li L. Chen X. Y. Zeng H. (2014). Leading dimensions in absorptive root trait variation across 96 subtropical forest species. New Phytol. 203, 863–872. 10.1111/nph.12842 24824672

[B35] Kramer-Walter K. R. Bellingham P. J. Millar T. R. Smissen R. D. Richardson S. J. Laughlin D. C. (2016). Root traits are multidimensional: specific root length is independent from root tissue density and the plant economic spectrum. J. Ecol. 104, 1299–1310. 10.1111/1365-2745.12562

[B36] Laliberté E. (2017). Below-ground frontiers in trait-based plant ecology. New Phytol. 213, 1597–1603. 10.1111/nph.14247 27735077

[B37] Leitner D. Klepsch S. Ptashnyk M. Marchant A. Kirk G. Schnepf A. (2010). A dynamic model of nutrient uptake by root hairs. New Phytol. 185, 792–802. 10.1111/j.1469-8137.2009.03128.x 20028467

[B38] Li H. Liu B. McCormack M. L. Ma Z. Guo D. (2017). Diverse belowground resource strategies underlie plant species coexistence and spatial distribution in three grasslands along a precipitation gradient. New Phytol. 216, 1140–1150. 10.1111/nph.14710 28758691

[B39] Liese R. Alings K. Meier I. C. (2017). Root branching is a leading root trait of the plant economics spectrum in temperate trees. Front.Plant Sci. 8, 315. 10.3389/fpls.2017.00315 28337213 PMC5340746

[B40] Lindahl B. D. Tunlid A. (2015). Ectomycorrhizal fungi–potential organic matter decomposers, yet not saprotrophs. New Phytol. 205, 1443–1447. 10.1111/nph.13201 25524234

[B41] Liu B. He J. Zeng F. Lei J. Arndt S. K. (2016). Life span and structure of ephemeral root modules of different functional groups from a desert system. New Phytol. 211, 103–112. 10.1111/nph.13880 26856386

[B42] Lopez-Bucio J. de la Vega O. M. Guevara-Garcia A. Herrera-Estrella L. (2000). Enhanced phosphorus uptake in transgenic tobacco plants that overproduce citrate. Nat. biotechnol. 18, 450–453. 10.1038/74531 10748530

[B43] Ma Z. Guo D. Xu X. Lu M. Bardgett R. D. Eissenstat D. M. (2018). Evolutionary history resolves global organization of root functional traits. Nature 555, 94–97. 10.1038/nature25783 29466331

[B44] Mao Z. Wang Y. McCormack M. L. Rowe N. Deng X. Yang X. (2018). Mechanical traits of fine roots as a function of topology and anatomy. Ann. Bot. 122, 1103–1116. 10.1093/aob/mcy076 29846521 PMC6324743

[B45] Marx D. H. (1972). Ectomycorrhizae as biological deterrents to pathogenic root infections. Annu. Rev. Phytopathol. 10, 429–454. 10.1146/annurev.py.10.090172.002241 18479192

[B46] McCormack M. L. Adams T. S. Smithwick E. A. Eissenstat D. M. (2012). Predicting fine root lifespan from plant functional traits in temperate trees. New Phytol. 195, 823–831. 10.1111/j.1469-8137.2012.04198.x 22686426

[B47] McCormack M. L. Dickie I. A. Eissenstat D. M. Fahey T. J. Fernandez C. W. Guo D. (2015). Redefining fine roots improves understanding of belowground contributions to terrestrial biosphere processes. New Phytol. 207, 505–518. 10.1111/nph.13363 25756288

[B48] McCormack M. L. Powell A. S. Iversen C. M. (2018). Better Plant Data at the Root of Ecosystem Models. EOS, Transactions American Geophysical Union (Online) 99. 10.1029/2018EO104093

[B49] McMurtrie R. E. Näsholm T. (2018). Quantifying the contribution of mass flow to nitrogen acquisition by an individual plant root. New Phytol. 218, 119–130. 10.1111/nph.14927 29226964

[B50] Meier I. C. Pritchard S. G. Brzostek E. R. McCormack M. L. Phillips R. P. (2015). The rhizosphere and hyphosphere differ in their impacts on carbon and nitrogen cycling in forests exposed to elevated CO2. New Phytol. 205, 1164–1174. 10.1111/nph.13122 25348688

[B51] Menge D. N. MacPherson A. C. Bytnerowicz T. A. Quebbeman A. W. Schwartz N. B. Taylor B. N. (2018). Logarithmic scales in ecological data presentation may cause misinterpretation. Nat. Ecol. Evol. 2, 1393. 10.1038/s41559-018-0610-7 30013132

[B52] Messier J. McGill B. J. Enquist B. J. Lechowicz M. J. (2017). Trait variation and integration across scales: is the leaf economic spectrum present at local scales? Ecography 40, 685–697. 10.1111/ecog.02006

[B53] Miller R. M. Jastrow J. D. Reinhardt D. R. (1995). External hyphal production of vesicular-arbuscular mycorrhizal fungi in pasture and tallgrass prairie communities. Oecologia 103, 17–23. 10.1007/BF00328420 28306940

[B54] Morris J. L. Puttick M. N. Clark J. W. Edwards D. Kenrick P. Pressel S. (2018). The timescale of early land plant evolution. Proc. Natl. Acad. Sci. U. S. A 115, E2274–E2283. 10.1073/pnas.1719588115 29463716 PMC5877938

[B55] Näsholm T. Högberg P. Franklin O. Metcalfe D. Keel S. G. Campbell C. (2013). Are ectomycorrhizal fungi alleviating or aggravating nitrogen limitation of tree growth in boreal forests? New Phytol. 198, 214–221. 10.1111/nph.12139 23356503

[B56] Nobel P. S. Schulte P. J. North G. B. (1990). Water influx characteristics and hydraulic conductivity for roots of Agave deserti Engelm. J. Exp. Bot. 41, 409–415. 10.1093/jxb/41.4.409

[B57] Orwin K. H. Kirschbaum M. U. St John M. G. Dickie I. A. (2011). Organic nutrient uptake by mycorrhizal fungi enhances ecosystem carbon storage: a model-based assessment. Ecol. Lett. 14, 493–502. 10.1111/j.1461-0248.2011.01611.x 21395963

[B58] Ostonen I. Püttsepp Ü. Biel C. Alberton O. Bakker M. R. Lõhmus K. (2007). Specific root length as an indicator of environmental change. Plant Biosyst. 141, 426–442. 10.1080/11263500701626069

[B59] Phillips R. P. Finzi A. C. Bernhardt E. S. (2011). Enhanced root exudation induces microbial feedbacks to N cycling in a pine forest under long-term CO2 fumigation. Ecol. Lett. 14, 187–194. 10.1111/j.1461-0248.2010.01570.x 21176050

[B60] R Core Development Team (2017). R: A Language and Environment for Statistical Computing. Austria: Vienna.

[B61] Raven J. Edwards D. (2001). Roots: evolutionary origins and biogeochemical significance. J. Exp. Bot. 52, 381–401. 10.1093/jxb/52.suppl_1.381 11326045

[B62] Raven J. A. Lambers H. Smith S. E. Westoby M. (2018). Costs of acquiring phosphorus by vascular land plants: patterns and implications for plant coexistence. New Phytol. 217, 1420–1427. 10.1111/nph.14967 29292829

[B63] Reich P. B. (2014). The world-wide ‘fast–slow’ plant economics spectrum: a traits manifesto. J. Ecol. 102, 275–301. 10.1111/1365-2745.12211

[B64] Roumet C. Birouste M. Picon-Cochard C. Ghestem M. Osman N. Vrignon-Brenas S. (2016). Root structure–function relationships in 74 species: evidence of a root economics spectrum related to carbon economy. New Phytol. 210, 815–826. 10.1111/nph.13828 26765311

[B65] Ryser P. (1996). The importance of tissue density for growth and life span of leaves and roots: A comparison of five ecologically contrasting grasses. Funct. Ecol. 10, 717–723. 10.2307/2390506

[B66] Schutzendubel A. Polle A. (2002). Plant responses to abiotic stresses: heavy metal-induced oxidative stress and protection by mycorrhization. J. Exp. Bot. 53, 1351–1365. 10.1093/jexbot/53.372.1351 11997381

[B67] Schweiger P. Robson A. Barrow N. (1995). Root hair length determines beneficial effect of a Glomus species on shoot growth of some pasture species. New Phytol. 131, 247–254. 10.1111/j.1469-8137.1995.tb05726.x

[B68] Smith S. E. Read D. J. (2008). Mycorrhizal symbiosis. London, UK: Academic Press.

[B69] Staddon P. L. Ramsey C. B. Ostle N. Ineson P. Fitter A. H. (2003). Rapid turnover of hyphae of mycorrhizal fungi determined by AMS microanalysis of 14C. Science 300, 1138–1140. 10.1126/science.1084269 12750519

[B70] Sun K. McCormack M. L. Li L. Ma Z. Guo D. (2016). Fast-cycling unit of root turnover in perennial herbaceous plants in a cold temperate ecosystem. Sci. Rep. 6, 19698. 10.1038/srep19698 26791578 PMC4726329

[B71] Talbot J. M. Martin F. Kohler A. Henrissat B. Peay K. G. (2015). Functional guild classification predicts the enzymatic role of fungi in litter and soil biogeochemistry. Soil Biol. Biochem. 88, 441–456. 10.1016/j.soilbio.2015.05.006

[B72] Taylor L. L. Leake J. R. Quirk J. Hardy K. Banwart S. A. Beerling D. J. (2009). Biological weathering and the long-term carbon cycle: integrating mycorrhizal evolution and function into the current paradigm. Geobiology 7, 171–191. 10.1111/j.1472-4669.2009.00194.x 19323695

[B73] Teste F. P. Marchesini V. A. Veneklaas E. J. Dixon K. W. Lambers H. (2018). Root dynamics and survival in a nutrient-poor and species-rich woodland under a drying climate. Plant Soil 424, 91–102. 10.1007/s11104-017-3323-9

[B74] Tjoelker M. G. Craine J. M. Wedin D. Reich P. B. Tilman D. (2005). Linking leaf and root trait syndromes among 39 grassland and savannah species. New Phytol. 167, 493–508. 10.1111/j.1469-8137.2005.01428.x 15998401

[B75] Treseder K. K. (2013). The extent of mycorrhizal colonization of roots and its influence on plant growth and phosphorus content. Plant Soil 371, 1–13. 10.1007/s11104-013-1681-5

[B76] Van Breemen N. Finlay R. Lundström U. Jongmans A. G. Giesler R. Olsson M. (2000). Mycorrhizal weathering: a true case of mineral plant nutrition? Biogeochemistry 49, 53–67. 10.1023/A:1006256231670

[B77] Vitousek P. M. Menge D. N. Reed S. C. Cleveland C. C. (2013). Biological nitrogen fixation: rates, patterns and ecological controls in terrestrial ecosystems. Philos Trans R Soc Lond B Biol Sci. 368, 20130119. 10.1098/rstb.2013.0119 23713117 PMC3682739

[B78] Weemstra M. Mommer L. Visser E. J. Ruijven J. Kuyper T. W. Mohren G. M. (2016). Towards a multidimensional root trait framework: a tree root review. New Phytol. 211, 1159–1169. 10.1111/nph.14003 27174359

[B79] Westoby M. Wright I. J. (2006). Land-plant ecology on the basis of functional traits. Trends Ecol. Evol. 21, 261–268. 10.1016/j.tree.2006.02.004 16697912

[B80] Wilson G. W. Hartnett D. C. (1998). Interspecific variation in plant responses to mycorrhizal colonization in tallgrass prairie. Am. J. Bot. 85, 1732–1738. 10.2307/2446507 21680333

[B81] Withington J. M. Reich P. B. Oleksyn J. Eissenstat D. M. (2006). Comparisons of structure and life span in roots and leaves among temperate trees. Ecol. Monogr. 76, 381–397. 10.1890/0012-9615(2006)076[0381:COSALS]2.0.CO;2

[B82] Wright I. J. Reich P. B. Westoby M. Ackerly D. D. Baruch Z. Bongers F. (2004). The worldwide leaf economics spectrum. Nature 428, 821–827. 10.1038/nature02403 15103368

[B83] Yanai R. D. Fahey T. J. Miller S. L. (1995). “Efficiency of nutrient acquisition by fine roots and mycorrhizae. Pages 75-103,” in Resource physiology of conifers: Acquisition, Allocation, and Utilization. Eds. Smith W. K. Hinckley T. M. (San Diego: Academic Press). 10.1016/B978-0-08-092591-2.50008-X

[B84] Zemunik G. Lambers H. Turner B. L. Laliberté E. Oliveira R. S. (2018). High abundance of non-mycorrhizal plant species in severely phosphorus-impoverished Brazilian campos rupestres. Plant Soil 424, 255–271. 10.1007/s11104-017-3503-7

[B85] Zemunik G. Turner B. L. Lambers H. Laliberté E. (2015). Diversity of plant nutrient-acquisition strategies increases during long-term ecosystem development. Nat. Plants 1, 15050. 10.1038/nplants.2015.50

[B86] Zhu Q. Iversen C. M. Riley W. J. Slette I. J. Vander Stel H. M. (2016). Root traits explain observed tundra vegetation nitrogen uptake patterns: implications for trait-based land models. J. Geophys. Res. Biogeosci. 121, 3101–3112. 10.1002/2016JG003554

