# Supplementary material for: Physical and Functional Constraints on Viable Belowground Acquisition Strategies
Source: Front Plant Sci. 2019 Oct 11;10:1215. doi: 10.3389/fpls.2019.01215 (PMC6797606; doi:10.3389/fpls.2019.01215)
Supplement: Supplementary file 1 [file DataSheet_1.docx]

**Supplemental Materials**

*Title:* **Physical and functional constraints on viable belowground acquisition strategies**

*Authors:* M. Luke McCormack and Colleen M. Iversen

**Text S1.** *Assembly and cleaning of data subset from the Fine-Root Ecology Database (FRED)*

Following download and initial cleaning of the FRED 2.4 dataset to remove unnecessary traits, auxiliary data, and data collected from mixed communities (i.e. not species-specific data), we conducted a series of additional cleaning and quality control steps to prepare the final dataset. First, two studies that reported their order-based root traits according to centrifugal ordering scheme (Pregitzer et al. 1997, Sun et al. 2016) while most other available data were reported according to the morphometric or centripetal ordering scheme (Fitter 1982). In these two cases, we reversed the order assessments such that the lowest order roots corresponded to the smallest most distal root in the fine-root system. This ensured that these data were directly comparable with the wider database. We removed trait data associated with pioneer first-order roots as opposed to fibrous first-order roots (Zadworny and Eissenstat 2011). Because of limited coverage and comparability, the few observations of bryophytes contained in FRED were removed from this analysis. All data not collected under outdoor, field conditions were removed (e.g. hydroponics and greenhouse studies; Poorter et al. 2016). However, data collected from field studies that included a fertilization treatment were retained. This represented less than 10% of the total data, of which less than half included nitrogen fertilizer. In all cases, these numbers were combined with non-treatment values and averaged into representative, single-species values (see below).

Classifications for mycorrhizal associations (e.g. arbuscular mycorrhizal, AM; ectomycorrhizal; EM; ericoid mycorrhizal ErM) were included in FRED when reported by the original study. For species that had not yet been classified, we added this information according to previously published studies and databases (Harley and Harley 1987, Brundrett and Kendrick 1988, Wang and Qiu 2006, Brundrett 2009, Maherali et al. 2016). However, in cases where a given species was nearly always reported as one mycorrhizal type but with a few exceptions reporting another mycorrhizal type (e.g. *Acer rubrum* commonly reported as AM but with a few observations of EM), we would only list the predominate association rather than listing the species as a dual mycorrhizal host. We then added additional data from the study by Li et al. (2017) that were originally unpublished and not available in FRED concerning fine-root diameter of first-order roots as well as mycorrhizal colonization rates across 26 grassland species. Finally, while some species’ traits in the dataset are represented by a single reported value, for many species, trait data were available from numerous independent observations (e.g. *Fraxinus mandshurica* and *Pinus sylvestris*). In all cases, within species trait data were averaged to yield a single trait value for a given trait and species. While this masks potential for intraspecific variation in fine-root traits (e.g. Zadworny et al. 2017), it serves a robust starting point for understanding broad patterns of fine-root trait variation across species.

The initial species average dataset based on species averages consisted of 374 individual plant species. However, data points for 5 species were removed as outliers due to extreme values based on visual inspection. The final dataset used here consisted of 369 species representing 76 families and 34 taxonomic orders. Of these 369 species, the majority were classified as associating with either AM or EM fungi with only 6 species being noted as primarily non-mycorrhizal (AM/NM) or fully non-mycorrhizal (NM). This dataset was used for regression analysis. An additional dataset in which all five traits (nitrogen content, diameter, specific root length-SRL, root tissue density-RTD, mycorrhizal colonization) were available for each species was also used for analysis and contained 117 individual species.

**Text S2.** *Parameterization of the heuristic model of belowground exploration efficiency.*

Data for root-specific traits were derived from a subset of the FRED 2.4 data used in the above analysis with specific emphasis placed on non-mycorrhizal and arbuscular mycorrhizal associates. We excluded ectomycorrhizal and ericoid mycorrhizal associates as well as dual-host mycorrhizal associates. The relationship between first-order root diameter (mm) and RTD (g cm^-3^) was expressed as:

$Root Tissue Density=0.476-0.6483*D+0.4184*D^2$ eq. 1

where *D* represents root diameter (mm). The relationship between first-order root diameter and percent mycorrhizal colonization was expressed as:

$Percent Colonization=0.7951+190.03*D-96.541*D^2$ eq. 2

We limited the root diameter range used in the heuristic model from 0.1 mm to 0.7 mm roots, reflecting a typical range of first-order root diameters observed in many terrestrial ecosystems (Pregitzer et al. 2002, Kong et al. 2014, Li et al. 2017). However, the empirical relationships described above were derived using FRED 2.4 as presented above, where first-order fine-root diameters ranged from *ca.* 0.09 to 1.1 mm. Initial data checks indicated that the relationship was similar regardless of whether it was derived using the full diameter range or a subset.

Data concerning traits of mycorrhizal fungi are not as widely reported as root traits which limits our ability to parameterize these traits as continuous variables. The values used here are intended to broadly represent relatively low and high values reported in the literature rather than a continuous empirical relationship with root diameter. Therefore, the model results should be viewed as potential boundaries for likely values seen in natural systems. Among the different scenarios, we included low and high estimates for hyphal diameter of 3 and 10 µm, respectively (Bååth and Söderström 1979, Miller et al. 1995). These are reflective of individual hyphae and not for aggregated hyphal rhizomorphs or cords. Low and high values for hyphal production rates, reported as the length of hyphae associated with a unit of colonized root length, were prescribed as 1 and 10 m of hyphal length per cm colonized root length (Abbott et al. 1984, Miller et al. 1995, Li et al. 2019). Finally, an average density of hyphal tissues was set to 1.1 g cm^-3^ and used for all fungal scenarios (Bakken and Olsen 1983), but we note that other studies have used lower estimates (e.g. 0.44 g cm-3; Yanai et al. 1995). Using these lower estimates does not qualitatively change our results; however, lower tissue densities for fungal hyphae would further exaggerate the patterns observed such that belowground exploration efficiency (surface area gains per construction cost) would see an even greater increase with greater reliance on fungal hyphae.

In total we present six different scenarios with the heuristic model. In addition to a scenario where only roots were considered (i.e. no contribution by mycorrhizal fungi), factorial combinations of high- and low hyphal production rates with high- and low first-order fine-root diameter were used in an additional four model scenarios (Table S1). We also present a ‘gradient’ scenario in which the hyphal production rate associated with roots increases with fine-root diameter such that the smaller-diameter roots derive a lower proportion of their absorptive surface area from fungal hyphae and the largest diameter roots are the most strongly associated with mycorrhizal fungi. This is consistent with studies reporting lower values of hyphal length or mycorrhizal dependency associated with sites or species with lower root diameters and higher SRL (Manjunath and Habte 1991, Miller et al. 1995, Liu et al. 2015), though exceptions have been observed within some sites where variation in hyphal length across species may be limited despite wide differences in root morphologies and mycorrhizal colonization rates (Chen et al. 2016). The concept of increased hyphal production rates associated with larger-diameter roots is further consistent with the idea that root cortical cells serve as the primary site of association and exchange between roots and arbuscular mycorrhizal fungi (Brundrett 2002). Therefore, as cortex volume increases in large-diameter roots (Kong et al. 2014), we may expect greater potential for exchange with plants to support growth of fungal hyphae.

**Figure S1.** Fine-root and fungal trait data used to parameterize the heuristic model. Smoothed-spline relationships are shown between first-order root diameter and mycorrhizal colonization (a; λ=0.05) and RTD (b, λ=0.05). These were then fit by a second-order polynomial to derive empirical relationships for model use. Data were initially taken from FRED 2.4 and then subset to include only species known to associate with arbuscular mycorrhizal fungi (as well as non-mycorrhizal species). Panel (c) depicts the high, low, and gradient hyphal production scenarios used to capture wide variability reported in literature.


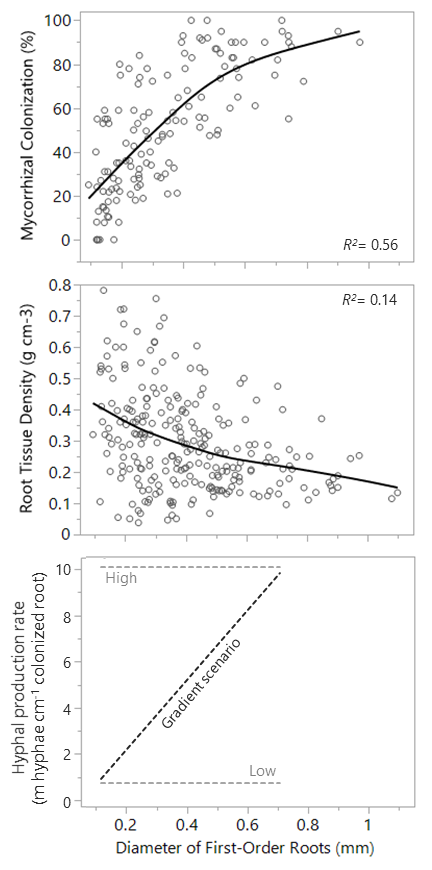


**Figure S2.** Correlations between mycorrhizal colonization and root diameter (panels a, b) or specific root length (SRL; panels c, d) separated by mycorrhizal association. Panels a and c present species that associate with arbuscular mycorrhizal fungi (AM), are considered facultatively AM (AMNM), or are considered non-mycorrhizal (NM). Panels b and d present only species that associate with ectomycorrhizal fungi (EM). Linear fits and Pearson’s correlation coefficient (*r*) are shown for significant relationships (*p* ≤ 0.05) along with sample numbers (*N*). Diameter and SRL data were log-transformed while mycorrhizal colonization was transformed using the angular transformation.

**
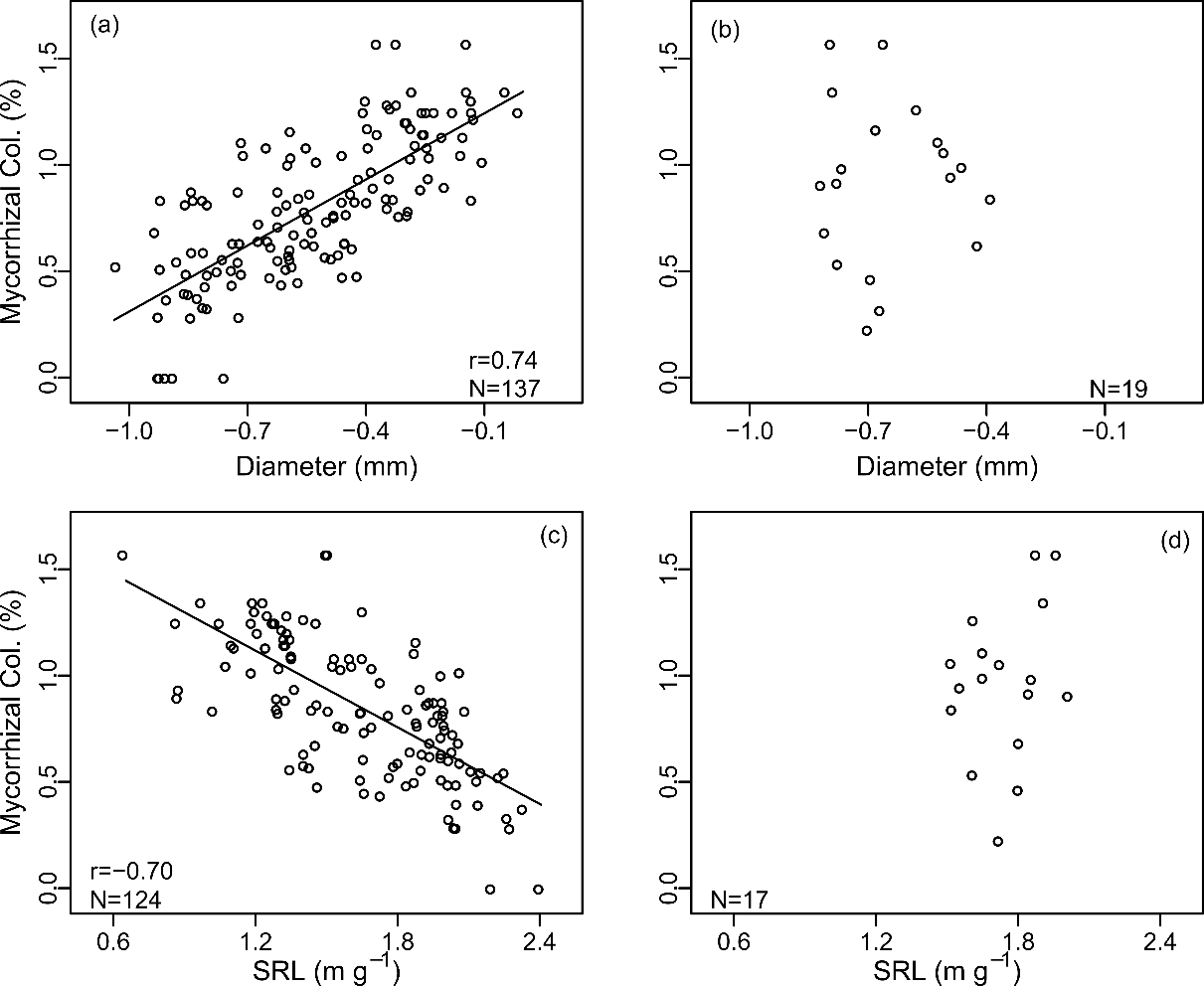
**

**Table S1**. Six general scenarios and fungal parameters used in the heuristic model of belowground exploration efficiency. Root parameters are based on empirical relationships and are reported in equations 1 and 2 and presented in Figure S1.

| **Scenario** | **Hyphal Tissue Density** (g cm^-3^)† | | | **Hyphal Diameter** (µm)‡ | | | **Hyphal Production Rate** (m hyphal length per cm colonized root length)* | | |
| --- | --- | --- | --- | --- | --- | --- | --- | --- | --- |
| *Fine roots only* | | NA | | NA | | 0 | | |  |
| Fine roots plus mycorrhizal hyphae | | |  |  |  | | |  |  |
| *Small diameter, low production* | | 1.1 | | 3 | | 1 | | |  |
| *Small diameter, high production* | | 1.1 | | 3 | | 10 | | |  |
| *Large diameter, low production* | | 1.1 | | 10 | | 1 | | |  |
| *Large diameter, high production* | | 1.1 | | 10 | | 10 | | |  |
| *Gradient of hyphal production* | | 1.1 | | 6.5 | | *y = 15(Root Diameter) - 0.5* | | |  |

†Bakken and Olsen (1983)

‡Bååth and Söderström (1979); Miller et al. (1995)

*Abbott et al. (1984); Miller et al. (1995); Li et al. (2019)

**Supplemental Citations**

Abbott, L., A. Robson, and G. d. Boer. 1984. The effect of phosphorus on the formation of hyphae in soil by the vesicular-arbuscular mycorrhizal fungus, *Glomus fasciculatum*. New Phytologist **97**:437-446.

Bååth, E., and B. Söderström. 1979. The significance of hyphal diameter in calculation of fungal biovolume. Oikos:11-14.

Bakken, L. R., and R. A. Olsen. 1983. Buoyant densities and dry-matter contents of microorganisms: conversion of a measured biovolume into biomass. Applied and Environmental Microbiology **45**:1188-1195.

Brundrett, M. C. 2002. Coevolution of roots and mycorrhizas of land plants. New Phytologist **154**:275-304.

Brundrett, M. C. 2009. Mycorrhizal associations and other means of nutrition of vascular plants: understanding the global diversity of host plants by resolving conflicting information and developing reliable means of diagnosis. Plant and Soil **320**:37-77.

Brundrett, M. C., and B. Kendrick. 1988. The mycorrhizal status, root anatomy, and phenology of plants in a sugar maple forest. Canadian Journal of Botany **66**:1153-1173.

Chen, W., R. T. Koide, T. S. Adams, J. L. DeForest, L. Cheng, and D. M. Eissenstat. 2016. Root morphology and mycorrhizal symbioses together shape nutrient foraging strategies of temperate trees. Proceedings of the National Academy of Sciences **113**:8741-8746.

Fitter, A. 1982. Morphometric analysis of root systems: application of the technique and influence of soil fertility on root system development in two herbaceous species. Plant, Cell & Environment **5**:313-322.

Harley, J. L., and E. Harley. 1987. A check-list of mycorrhiza in the British flora. New Phytologist:1-102.

Kong, D. L., C. G. Ma, Q. Zhang, L. Li, X. Y. Chen, H. Zeng, and D. L. Guo. 2014. Leading dimensions in absorptive root trait variation across 96 subtropical forest species. New Phytologist **203**:863-872.

Li, H., B. Liu, M. L. McCormack, Z. Ma, and D. Guo. 2017. Diverse belowground resource strategies underlie plant species coexistence and spatial distribution in three grasslands along a precipitation gradient. New Phytologist **216**:1140-1150.

Li, L., M. L. McCormack, F. Chen, H. Wang, Z. Ma, and D. Guo. 2019. Different responses of absorptive roots and arbuscular mycorrhizal fungi to fertilization provide diverse nutrient acquisition strategies in Chinese fir. Forest Ecology and Management **433**:64-72.

Liu, B., H. Li, B. Zhu, R. T. Koide, D. M. Eissenstat, and D. Guo. 2015. Complementarity in nutrient foraging strategies of absorptive fine roots and arbuscular mycorrhizal fungi across 14 coexisting subtropical tree species. New Phytologist **208**:125-136.

Maherali, H., B. Oberle, P. F. Stevens, W. K. Cornwell, D. J. McGlinn, M. E. Frederickson, and A. A. Winn. 2016. Mutualism Persistence and Abandonment during the Evolution of the Mycorrhizal Symbiosis. American Naturalist **188**:E113-E125.

Manjunath, A., and M. Habte. 1991. Root morphological characteristics of host species having distinct mycorrhizal dependency. Canadian Journal of Botany **69**:671-676.

Miller, R. M., J. D. Jastrow, and D. R. Reinhardt. 1995. External hyphal production of vesicular-arbuscular mycorrhizal fungi in pasture and tallgrass prairie communities. Oecologia **103**:17-23.

Poorter, H., F. Fiorani, R. Pieruschka, T. Wojciechowski, W. H. Putten, M. Kleyer, U. Schurr, and J. Postma. 2016. Pampered inside, pestered outside? Differences and similarities between plants growing in controlled conditions and in the field. New Phytologist **212**:838-855.

Pregitzer, K. S., J. L. DeForest, A. J. Burton, M. F. Allen, R. W. Ruess, and R. L. Hendrick. 2002. Fine root architecture of nine North American trees. Ecological Monographs **72**:293-309.

Pregitzer, K. S., M. E. Kubiske, C. K. Yu, and R. L. Hendrick. 1997. Relationships among roof branch order, carbon, and nitrogen in four temperate species. Oecologia **111**:302-308.

Sun, K., M. L. McCormack, L. Li, Z. Ma, and D. Guo. 2016. Fast-cycling unit of root turnover in perennial herbaceous plants in a cold temperate ecosystem. Scientific reports **6**:19698.

Wang, B., and Y. L. Qiu. 2006. Phylogenetic distribution and evolution of mycorrhizas in land plants. Mycorrhiza **16**:299-363.

Yanai, R. D., T. J. Fahey, and S. L. Miller. 1995. Efficiency of nutrient acquisition by fine roots and mycorrhizae. Pages 75-103 *in* W. K. Smith and T. M. Hinckley, editors. Resource physiology of conifers: Acquisition, Allocation, and Utilization. Academic Press, San Diego.

Zadworny, M., and D. M. Eissenstat. 2011. Contrasting the morphology, anatomy and fungal colonization of new pioneer and fibrous roots. New Phytologist **190**:213-221.

Zadworny, M., M. L. McCormack, R. Żytkowiak, P. Karolewski, J. Mucha, and J. Oleksyn. 2017. Patterns of structural and defense investments in fine roots of Scots pine (Pinus sylvestris L.) across a strong temperature and latitudinal gradient in Europe. Global Change Biology **23**:1218-1231.
